# Exercising Caution Upon Waking–Can Exercise Reduce Sleep Inertia?

**DOI:** 10.3389/fphys.2020.00254

**Published:** 2020-04-07

**Authors:** Katya Kovac, Sally A. Ferguson, Jessica L. Paterson, Brad Aisbett, Cassie J. Hilditch, Amy C. Reynolds, Grace E. Vincent

**Affiliations:** ^1^Appleton Institute, School of Health, Medical and Applied Sciences, Central Queensland University, Adelaide, SA, Australia; ^2^Institute for Physical Activity and Nutrition (IPAN), School of Exercise and Nutrition Sciences, Deakin University, Geelong, VIC, Australia; ^3^Fatigue Countermeasures Laboratory, San José State University Research Foundation, Moffett Field, CA, United States

**Keywords:** exercise, sleep inertia, waking, cortisol awakening response, thermoregulation, cerebral blood flow, functional connectivity

## Abstract

Sleep inertia, the transitional state of reduced alertness and impaired cognitive performance upon waking, is a safety risk for on-call personnel who can be required to perform critical tasks soon after waking. Sleep inertia countermeasures have previously been investigated; however, none have successfully dissipated sleep inertia within the first 15 min following waking. During this time, on-call personnel could already be driving, providing advice, or performing other safety-critical tasks. Exercise has not yet been investigated as a sleep inertia countermeasure but has the potential to stimulate the key physiological mechanisms that occur upon waking, including changes in cerebral blood flow, the cortisol awakening response, and increases in core body temperature. Here, we examine these physiological processes and hypothesize how exercise can stimulate them, positioning exercise as an effective sleep inertia countermeasure. We then propose key considerations for research investigating the efficacy of exercise as a sleep inertia countermeasure, including the need to determine the intensity and duration of exercise required to reduce sleep inertia, as well as testing the effectiveness of exercise across a range of conditions in which the severity of sleep inertia may vary. Finally, practical considerations are identified, including the recommendation that qualitative field-based research be conducted with on-call personnel to determine the potential constraints in utilizing exercise as a sleep inertia countermeasure in real-world scenarios.

## Introduction

In an early investigation, Grotjahn (1942, p. 3) wrote the following about waking from sleep: “the sleeper is abruptly turned toward reality, tries to catch the situation but is not immediately ready for a proper perception of reality”. It is now known that waking from sleep is a transitional process in which the restoration of complete alertness and cognitive performance takes place over a period of time (Trotti, 2016). Grotjahn’s early observation that we are “not immediately ready for a proper perception of reality” is an accurate description of this transitory state between sleep and full alertness, which has since been termed “sleep inertia” (Lubin et al., 1976).

Sleep inertia is the temporary state of reduced alertness and impaired cognition found upon waking (Tassi and Muzet, 2000). During sleep inertia, impairments have been found in various cognitive domains such as working memory (Signal et al., 2012), vigilance (Dinges et al., 1985), visual search (Burke et al., 2015), logical reasoning (Naitoh et al., 1993), and decision making (Naitoh et al., 1993; Bruck and Pisani, 1999). Typically, sleep inertia takes between 15 and 30 min to dissipate with performance improving over time (Tassi and Muzet, 2000; Trotti, 2016). Some studies, however, have found that performance may not plateau or stabilize until 2 h post-waking, even after a nominally sufficient sleep opportunity (e.g., 8-h nocturnal sleep) (Jewett et al., 1999). The duration and/or severity of sleep inertia can be increased by a number of factors including (a) waking during the night (Dinges et al., 1985; Scheer et al., 2008; Silva and Duffy, 2008); (b) waking from slow wave sleep (Feltin and Broughton, 1968; Wilkinson and Stretton, 1971; Tassi et al., 2006); and (c) prior sleep loss (Dinges et al., 1985; Tassi et al., 2006; McHill et al., 2017, 2019). The negative impacts of sleep inertia are particularly relevant to workers.

The 24-h demands of modern society mean that an increasing number of workers are not afforded the luxury of time to properly “wake up” before going about their day. This is particularly salient for personnel with on-call arrangements. On-call or “stand-by” arrangements are often utilized to cover periods of work where the demand is typically low (e.g., at night) and unpredictable (e.g., emergency scenarios) (Ferguson et al., 2016). During on-call shifts, workers are on stand-by but, if called, may be required to respond immediately (Nicol and Botterill, 2004; Hall et al., 2016b). Workers’ responses to call-outs can include a range of actions from taking a call and providing advice from home to traveling to an emergency site. Metropolitan firefighters who are called while in the station are required to don their safety equipment and get into their vehicle within 90 s of receiving a call (Paterson et al., 2016). Calls received at night have the potential to wake workers from sleep, with implications for time and safety-critical industries such as emergency services and healthcare (Lawrence et al., 2018). Sleep inertia can negatively affect not only the worker but also the safety of co-workers, civilians on the roads, and individuals being attended to during emergency scenarios.

Given the safety risks that sleep inertia presents, a number of studies have investigated potential countermeasures to minimize the duration and severity of sleep inertia (Hilditch et al., 2016). Proactive countermeasures (i.e., countermeasures performed prior to waking), such as coffee consumption prior to sleep, have been shown to successfully eliminate sleep inertia after 2-h naps (Van Dongen et al., 2001). However, such strategies may not be practical for on-call workers where awakenings are unpredictable. Reactive countermeasures to sleep inertia (i.e., countermeasures implemented upon waking) may thus be more suitable in the on-call context. Reactive countermeasures that have been investigated include caffeine ingestion upon waking (Newman et al., 2013), light exposure (Hayashi et al., 2003; Santhi et al., 2013), sound manipulation (Tassi et al., 1992; Hayashi et al., 2004), body temperature manipulation (Kräuchi et al., 2004; Krauchi et al., 2006), and face washing (Hayashi et al., 2003). To date, there is limited evidence that these countermeasures can successfully dissipate sleep inertia within 15 min post-waking. Since some on-call roles require a response within minutes, countermeasures that do not meaningfully improve performance (e.g., minutes after waking) cannot be relied upon. As a result, further research into reactive countermeasures that dampen sleep inertia within the critical first minutes after waking is urgently needed.

A potential sleep inertia countermeasure that has been proposed, but not yet tested, is performing exercise upon waking (Hilditch et al., 2016). The purported efficacy of exercise in the minutes following waking is borne from its stimulation of many of the physiological mechanisms that are suppressed upon waking. These processes include the reactivation and deactivation of various brain regions and changes in cerebral blood flow (CBF) (Balkin et al., 2002), the cortisol awakening response (CAR) (Clow et al., 2010), and thermoregulatory processes [e.g., increases in core body temperature (CBT) and cooling of extremities] (Kräuchi et al., 2004). Exercise, especially aerobic exercise, is well known to activate each of these physiological processes (Kindermann et al., 1982; Shellock et al., 1985; Kubitz and Mott, 1996; Ide and Secher, 2000; Nielsen et al., 2001; Drust et al., 2005; Hill et al., 2008; Crewther et al., 2010; Rajab et al., 2014; Anderson and Wideman, 2017). If exercise can speed up the physiological changes which occur upon waking, it may influence the duration and severity of sleep inertia and could be a suitable reactive countermeasure. The physiological underpinnings of waking, including the changes that occur to the brain, cortisol levels, and CBT are discussed further in the following section. Following a description of each process, an explanation of how exercise can stimulate or accelerate that process is provided. To facilitate comparisons between studies within the following review, the concept of sleep inertia is defined as the phenomena observed during the transition from sleep to wake (Tassi and Muzet, 2000).

## The Physiology of Waking

### Cerebral Blood Flow and Brain Activation

#### Upon Waking

Cerebral blood flow is the volume of blood supplied to the brain at any given time (Fantini et al., 2016). CBF ensures delivery of oxygen to the brain, enabling metabolism and the removal of metabolic waste products (Fantini et al., 2016). During sleep, CBF is typically reduced below waking levels (Hajak et al., 1994). Balkin et al. (2002) utilized positron-emission tomography (PET) scans of the brain to investigate CBF during the process of waking. Within 5 min of waking, blood flow is most prominent in the thalamus and brainstem, and the reactivation of these areas specifically promotes consciousness (Balkin et al., 2002). The reinstatement of alertness (the ability to concentrate, focus on a task, and be motivated) (Shapiro et al., 2006) occurs much later (20–30 min post-waking). This aligns with CBF reaching waking levels in the anterior-cortical regions of the brain, such as the prefrontal cortex, which is involved in cognitive processes. The time course of progressive return of CBF to waking levels in the anterior-cortical regions of the brain (i.e. alertness) coincides with the time course of sleep inertia dissipation.

Previous research has demonstrated that electroencephalographic (EEG) estimates of neural activity immediately post-waking is characterized by an increase in low-frequency oscillatory power and is different to presleep EEG power (Ferrara et al., 2006). In addition, upon waking, oscillatory power within the beta range is suppressed, an oscillatory pattern that is normally associated with an alert state; moreover, the spatial specificity of this pattern suggests a large role for frontal brain regions, which are generally associated with higher-level cognitive processing (Ferrara et al., 2006; Marzano et al., 2011; Vallat et al., 2019). Studies estimating functional connectivity, which estimates the statistical dependency of neural activity between regions of the brain, show that upon waking, these connectivity patterns exhibit similar traits to those observed during both stage 2 and slow wave sleep and, are significantly different to connectivity estimated 25 min post-waking (Vallat et al., 2019). For example, at 5 min post-waking, Vallat et al. (2019) found increases in connectivity in many large networks of the brain, specifically between the default mode network and the dorsal attention network and between the salience network and the sensory motor network. These connectivity changes also occur when sleeping, compared to a baseline rest period (Vallat et al., 2019). However, at 25 min post-waking, decreases in connectivity between these same networks are seen, possibly representing an optimal waking function (Vallat et al., 2019). Thus, decreases in CBF, brain activity, and changes in functional connectivity coincide with the sleep inertia window. Therefore, in the early stages of waking, sleep inertia could potentially be a result of the continuation of sleep-like brain activity as measured by functional connectivity.

#### During Exercise

Exercise increases blood flow to a large proportion of the brain, ensuring sufficient blood supply and oxygen consumption for brain metabolism (Ide and Secher, 2000; Querido and Sheel, 2007). CBF has been shown to increase in response to both high-intensity (13% increase) and low-intensity (6% increase) exercises (Williamson et al., 1999) and also in response to very short maximal exercises (i.e., maximal sprints; ∼16% increase) (Curtelin et al., 2018). Increases in CBF in response to exercise are triggered by an increase in brain metabolism (Querido and Sheel, 2007) and occur as a result of an increase in the partial pressure of carbon dioxide which causes vasodilation in cerebral vessels (Betz, 1972).

Alongside increases in CBF, exercise can also increase functional connectivity in areas of the brain associated with various aspects of cognitive processing (Li et al., 2014; Rajab et al., 2014; Weng et al., 2017). Using resting state functional magnetic resonance imaging (rs-fMRI), Rajab et al. (2014) found no change in connectivity within the default mode network after 20 min of exercise compared to pre-exercise. However, an increase in connectivity in brain regions responsible for the sense of touch and the relay of motor and sensory information was observed post-exercise when compared to pre-exercise (Rajab et al., 2014). In addition, other studies have found an enhanced integration in the brain networks involved with learning and memory, executive control, and attention after 30 min of moderate-intensity exercise (Weng et al., 2017). This was accomplished by comparing the spatial similarities between a seed-based functional connectivity analyses and predefined functional networks (Weng et al., 2017). These findings by Weng et al. (2017) illustrate that connectivity in areas of the brain not directly related to movement can also be affected by exercise. Increased cortical activity in areas responsible for complex decision making and visual processing was also found during a working memory task which was preceded by 20 min of moderate-intensity exercise (Li et al., 2014). Exercise of shorter durations (<5 min) also increases cortical activity at both low (Mechau et al., 1998) and moderate (Kubitz and Mott, 1996; Nielsen et al., 2001) intensities. Further, exercise results in increased cortical activity in the regions of the brain responsible for the processing of sensory information, language processing, and visual processing (Mechau et al., 1998; Nielsen et al., 2001).

Since acute exercise has been shown to increase cerebral activity, it is unsurprising that studies have found that acute exercise may improve cognitive function. Reviews of the literature have found a small positive effect of acute exercise on cognition (Etnier et al., 1997; Lambourne and Tomporowski, 2010; Chang et al., 2012). The effects of exercise on cognition, however, are dependent on a number of moderating factors including the duration and intensity of the exercise (Chang et al., 2012). A meta-analysis by Chang et al. (2012) found a small positive effect (*d* = 0.097) of acute exercise on cognitive performance and examined specific moderators (e.g., duration of exercise and exercise intensity). “Hard” or “very hard” exercise had a positive effect on cognitive performance when tested at least 1 min post-exercise (Chang et al., 2012). While there was no effect of short durations of exercise on cognitive performance, interactions between exercise duration and intensity, and cognitive performance were not examined. Improvements in memory storage, memory retrieval, and speeded mental processes following exercise have also been observed (Lambourne and Tomporowski, 2010). Interestingly, the exercise mode was found to moderate cognitive performance, with better performance found during and following cycling compared to treadmill running (Lambourne and Tomporowski, 2010). The authors suggest that higher energy metabolism and sensory processing are associated with running compared to cycling, and this may interfere with cortical activation and reduce efficiency in cognitive processing (Lambourne and Tomporowski, 2010).

The positive effects of exercise on cognitive performance may be due to an increase in the release of catecholamines: adrenaline, noradrenaline, and dopamine (Tomporowski, 2003; Davranche and Audiffren, 2004; Lambourne and Tomporowski, 2010). These catecholamines, specifically noradrenaline and dopamine, are involved in activating areas of the brain responsible for cognitive functions such as working memory, reaction time, and learning (McMorris, 2009). A significant correlation between plasma adrenaline levels and reaction time during exercise has been found (Chmura et al., 1994); however, evidence of a direct relationship between catecholamine concentration and cognitive performance during exercise is equivocal, and more research is needed in this area (McMorris et al., 2008; McMorris, 2009). While further exploration of this concept is outside the scope of the present review, the inclusion of measurements of catecholamines during exercise is discussed later as a consideration for future research. Overall, the existing literature suggests that exercise, even of a short duration and at moderate to high intensities, could be a suitable strategy to stimulate increases in CBF and functional connectivity within the brain upon waking and, by extension, to target the cognitive performance deficits associated with sleep inertia.

### Cortisol Awakening Response

#### Upon Waking

Increases in CBF and the changes in brain activity upon waking also coincide with, and potentially influence, another waking physiological process: the CAR. Cortisol is the hormonal product of the hypothalamic–pituitary–adrenal (HPA) axis. The HPA axis is the central stress response system and is involved in an increase in metabolism and energy mobilization during the stress response (Kudielka and Kirschbaum, 2005). The secretion of cortisol also occurs throughout the day and follows a circadian cycle with an early morning peak and then a decline throughout the day with the lowest secretion levels around midnight (Kirschbaum and Hellhammer, 1989). While cortisol secretion exhibits a circadian rhythm, the CAR is a markedly separate phenomenon in which cortisol concentrations sharply increase by between 50 and 160% within 30–45 min post-waking and decline thereafter (Pruessner et al., 1997; Clow et al., 2004).

Upon waking, the initiation of the CAR appears to temporally correspond with (a) the return of consciousness and (b) the blood flow to the relevant areas in the brain (Clow et al., 2010). The cortisol peak, which happens approximately 30–45 min post-waking, temporally corresponds with the dissipation of sleep inertia (Tassi and Muzet, 2000). As such, the CAR may influence sleep inertia dissipation, with the peak of the CAR partially responsible for the return to alertness upon waking (Clow et al., 2010). Indeed, Pruessner et al. (1997, p. 2548) suggested that the purpose of the CAR was likely to “provide the organism to shift from a resting to an active state”. Other studies have also found positive associations between manipulated increases in cortisol levels using orally ingested cortisol with subjective (Tops et al., 2006) and objective measures of alertness (Abercrombie et al., 2005). The magnitude of the CAR can be influenced by a number of factors including prior or anticipatory (next-day) stress (Rohleder et al., 2007; Fries et al., 2009). Sleep-related factors, such as prior sleep duration and stage of sleep upon waking [rapid eye movement (REM) or non-REM] (Wilhelm et al., 2007), can also influence the CAR. These same factors have also been shown to influence sleep inertia (Tassi and Muzet, 2000). As such, there is evidence to suggest that the CAR is potentially one of the physiological mechanisms which drive sleep inertia, although further research into this link is needed.

#### During Exercise

There is limited research on how exercise acutely impacts the CAR. To date, studies which have examined the effect of acute exercise on the CAR have only investigated exercise performed during the evening (Garde et al., 2009; Anderson and Wideman, 2017; Ucar et al., 2018). These studies found that a single bout of exercise performed in the evening had no impact on the CAR (Garde et al., 2009; Ucar et al., 2018). Exercise has not been performed in close proximity to the CAR, and to the authors’ knowledge, there have not been any investigations into the impact of acute exercise performed immediately upon waking on the CAR.

Despite the limited research on the effect of acute exercise on the CAR, some evidence suggests that acute exercise does affect circulating cortisol levels (Kindermann et al., 1982; Hill et al., 2008; Crewther et al., 2010; Anderson and Wideman, 2017). This is one of the reasons that it is recommended for study participants to remain sedentary when cortisol samples are obtained (Stalder et al., 2016). Further, changes in circulating cortisol levels as a result of exercise may be dependent on exercise intensity (Hill et al., 2008). Compared to before exercise, cortisol levels are significantly increased after 30 min of cycling exercise at 60 and 80% of maximal oxygen intake (VO_2_ max) but not at resting or after 30 min of cycling exercise at 40% VO_2_ max (Hill et al., 2008). These findings suggest that moderate- to high-intensity exercise, but not low-intensity exercise, may increase cortisol levels. Further, ultra-short bursts of high-intensity exercise can increase circulating cortisol levels. For example, following 1.5-min high-intensity and 30-s maximum-intensity cycling, cortisol levels have been shown to increase by 35 and 63%, respectively, above resting values (Kindermann et al., 1982; Crewther et al., 2010). These studies demonstrate the efficacy of an ultra-short bout of acute exercise in increasing circulating cortisol levels. Thus, findings on the effect of acute exercise on circulating cortisol levels at times of the day other than waking support the hypothesis that a short burst of exercise performed upon waking has the potential to increase the magnitude of the CAR. An increase in CAR magnitude could, in turn, increase alertness and reduce the duration and severity of sleep inertia. Further investigations in controlled studies are needed.

### Thermoregulation

#### Upon Waking

Thermoregulation, the maintenance of body temperature, may also play a role in sleep inertia. In humans, body temperature is differentially regulated by the outer shell, composed of the skin and subcutaneous muscles and tissues, and the core, composed of cranial, thoracic, and abdominal cavities (Gisolfi et al., 2000). During sleep, CBT gradually reduces and heat travels to the extremities (e.g., hands and feet) through distal vasodilation to promote heat loss and good-quality sleep (Kräuchi et al., 2004). The reverse of this process occurs when waking from both an overnight sleep (Kleitman et al., 1938) and a nap (Kräuchi et al., 2004). In a study of thermoregulation upon waking, Kräuchi et al. (2004) found that soon after waking, vasoconstriction of extremities occurs, preventing further heat loss and retaining body heat in the core. In addition, a decrease in subjective sleepiness with increasing CBT post-waking suggests that CBT changes could play a part in the dissipation of sleep inertia and in the return to normal waking alertness levels (Kräuchi et al., 2004).

The effect of body temperature on cognitive performance is well established, with variations in reaction time coinciding with the peaks and troughs of diurnal body temperature (Kleitman et al., 1938). Poorer cognitive performance was observed at night and early in the morning (when body temperature is generally at its lowest) and improved performance in the afternoon (Kleitman et al., 1938). More recently, a 28-h forced desynchrony protocol reported the influence of CBT on performance and alertness, independent of circadian phase and sleep pressure (Wright et al., 2002). There was an association between elevated CBT and better performance on visual attention, reaction time, and working memory tasks, as well as greater subjective alertness across the entire study period (Wright et al., 2002). Based on these findings, Kräuchi et al. (2004) suggested that a period of “inertia” may occur even in the absence of sleep if distal vasodilation and a drop in CBT are induced. This may occur after extended periods of relaxation-type activities such as meditating, taking a hot bath, or even lying down and resting without sleeping (Kräuchi et al., 2004). This possibility could be particularly relevant for on-call workers with capacity in their work arrangements to rest and relax when on call or during quiet periods.

The current research on thermoregulation and cognitive performance, as well as subjective sleepiness, suggests a link between thermoregulation and sleep inertia. The return to alertness post-waking may result from the body’s need to achieve a CBT which is optimum for arousal and therefore cognitive performance. Since CBT is generally lowest prior to waking, this may explain why it takes time for performance impairments to dissipate during sleep inertia. If the rate of sleep inertia dissipation is influenced by CBT, then manipulations to increase core temperature could promote a faster dissipation process.

#### During Exercise

It has long been established that muscular exercise induces an increase in CBT, which then triggers thermoregulatory processes to balance heat production and heat loss (Nielsen, 1938) as cited by Sawka et al. (2010). The increases in CBT found during exercise are a consequence of muscular metabolism to supply the energy required for muscular function (Kenny and Flouris, 2014). The heat produced during muscular metabolism is transferred from the muscles to the blood and the tissues surrounding the muscles, leading to an increase in CBT (Kenny et al., 2003). Increases in CBT occur rapidly at the initiation of exercise (Takeda and Okazaki, 2018). The degree of CBT increase is dependent on the intensity of the exercise, with higher-intensity exercise leading to a greater increase in heat production and thus larger CBT increases (Nielsen, 1938).

Little is known about the effect of a short bout of exercise on CBT immediately post-exercise, or when performed upon waking. Data from the available literature suggest that intermittent high-intensity cycle exercise (15-s high-intensity cycling followed by 15-s rest) performed for 5 min can increase CBT by 0.5°C (Drust et al., 2005). Moderate-intensity cycle exercise performed for 6 min has also been found to increase CBT by 0.2°C (Shellock et al., 1985), and ultra-short bouts of maximal exercise (5-s bouts) produced smaller increases (∼0.1°C) (Cochrane et al., 2008). Improvements to cognitive performance have been found with increases in CBT of 0.15°C (Wright et al., 2002); therefore, even smaller increases in CBT of 0.1°C may be meaningful when attempting to counter sleep inertia. In considering the available literature, a short duration of exercise may augment the increase in CBT observed upon waking and, thus, could accelerate the dissipation of sleep inertia.

## Could Exercise Performed Upon Waking Reduce Sleep Inertia?

The initiation of three physiological processes upon waking (increases in CBF, CAR, and CBT) parallel the decay of sleep inertia. It has been demonstrated that brief exercise bouts can accelerate each of these physiological responses, and this evidence highlights the potential for exercise upon waking to be a sleep inertia countermeasure. The following section will present considerations for (a) research investigating whether exercise performed upon waking can reduce sleep inertia (summarized in Table 1) and (b) practitioners and workers looking to implement sleep inertia countermeasures in the field.

**TABLE 1:** Experimental and practical considerations for sleep inertia countermeasure research.

Experimental consideration	Practical importance
Exercise intensity	Determine the intensity that workers need to exercise upon waking to counter sleep inertia.
Exercise duration	Determine if a short duration (<2 min) of exercise can counter sleep inertia.
Inclusion of physiological measures (CAR, CBT, CBF, neural activity)	Determine if exercise produces a significant change in physiology upon waking compared to no exercise.
Sleep conditions: • Time of day • Prior sleep restriction • Sleepdepth	Determine the conditions under which exercise performed upon waking is effective or ineffective in countering sleep inertia.
Combination of exercise with other proposed countermeasures (e.g., caffeine)	Determine whether workers should utilize a single or combined strategy to better counter sleep inertia.
Inclusion of subjective and objective measures of sleep inertia	Determine the effect of exercise on alertness as perceived (subjective) vs. the impact on tangible performance benefits (objective).
Inclusion of measures of subsequent sleep and circadian phase	Determine whether exercise, when used as a sleep inertia countermeasure, impacts workers’ subsequent sleep and circadian phase.
Controlling for individual differences (chronotype, age, and sex)	Consider and control for the impact of differences in chronotype, age, and sex on sleep inertia to better determine the effectiveness of exercise as a sleep inertia countermeasure for different individuals.
Influence of type of waking	Determine the effectiveness of exercise as a sleep inertia countermeasure when workers are abruptly woken compared to when they are gently woken and when responding to critical events (e.g., emergencies) compared to when responding to non-critical events (e.g., false alarms).

*CAR = cortisol awakening response, CBT = core body temperature, and CBF = cerebral blood flow.*

### Considerations for Researchers

#### Exercise Intensity

In order to provide appropriate recommendations for implementation, it is necessary to determine whether the effect of exercise on sleep inertia varies with intensity. Under controlled laboratory conditions, the potential dose-dependent effect of exercise intensity on sleep inertia could be determined by implementing different exercise intensities (e.g., light, moderate, and high intensities) upon waking while measuring sleep inertia and comparing results to a sedentary condition. Different exercise intensities could be monitored by utilizing exercise ergometers (e.g., cycle ergometers) which can measure work performance. It is also important that standardized protocols are followed to allow meaningful comparisons between studies. For example, the parameters for sedentary, light-intensity, moderate-intensity, vigorous, and high-intensity exercises defined in the position statement on physical activity and exercise intensity terminology by Norton et al. (2010) would be appropriate. Once the ideal intensity of exercise is determined, future research may also consider investigating the type of exercise employed and whether exercise requiring limited equipment (e.g., running on the spot and jumping jacks) is effective, especially given that on-call workers may not always have access to exercise equipment at home or in the workplace. While outside the scope of this present review, further review and research is needed to determine whether different types of exercise, for example, resistance exercise, are equally effective as sleep inertia countermeasures. This information may be useful given that different types of exercise may be more practical to integrate in the workplace than others.

#### Exercise Duration

Exercise performed as a sleep inertia countermeasure needs to be of a short duration to limit interference or delay in the work of on-call personnel. As a result, it is important that future research determines whether short durations of exercise upon waking are capable of reducing both the duration and severity of sleep inertia. Experimental investigations into the effect of exercise on sleep inertia for on-call workers should therefore limit exercise to durations that are practical for workers. Given the time-critical nature of on-call work, it is possible that for some emergency scenarios, a delay in response of 30 s may be too long. As such, researchers should determine the minimum duration of exercise necessary to meaningfully counter sleep inertia. Alternatively, longer durations of exercise (e.g., up to 10 min) may still be practical and beneficial for workers who have designated nap breaks at work and can factor a recovery period into their schedule before returning to work (e.g., pilots and truck drivers). Longer durations of exercise, potentially of up to 10 min in duration, should also be investigated for use with workers who have predictable nap opportunities.

#### Inclusion of Physiological Measures

Given the associations outlined in this review, experimental investigations into the effect of exercise on sleep inertia should also include measures of CBF, functional connectivity, the CAR, and CBT. This will help determine whether exercise upon waking does elicit a change in physiology. Physiological measurements should be taken immediately upon waking, as well as after the exercise protocol, and should be compared to measurements taken at an equivalent time in a sedentary control condition to allow for comparisons between exercise and no-exercise conditions. CBF upon waking and post-exercise could be measured using PET scans and EEG. Where access to PET equipment is not possible, measurement of brain activity via EEG pre- and post-exercise is achievable and has been successfully undertaken in previous research (Bailey et al., 2008). The CAR can be measured non-invasively by collecting saliva samples upon waking and at 15, 30, 45, and 60 min post-waking (Stalder et al., 2016). Relatively new technology has also reduced the invasiveness of CBT measurement with telemetric pills, which have been validated against traditional measures of CBT (Byrne and Lim, 2007). Comparing the time course of both physiological and cognitive responses to exercise upon waking might also help to better understand the relationship between each physiological aspect of waking and sleep inertia. Further, physiological mechanisms, such as increases in catecholamines (adrenaline, noradrenaline, and dopamine) may be involved in or mediate the link between exercise and alertness and also warrant further investigation. It should also be noted that while these physiological processes have been discussed separately in this review, it is possible that there are inter-dependencies between each of the processes, given the temporal overlap of their occurrences. For example, Clow et al. (2010) propose that the changes in the brain that occur upon waking may initiate the CAR. It is also possible that the CAR may interact with the increase in CBT found upon waking, which, in turn, is likely to differ depending on the circadian phase of CBT. More research into the inter-dependencies of these physiological processes is needed.

#### Sleep Conditions (Time of Day, Prior Sleep Restriction, and Sleep Depth)

As described, a number of factors influence sleep inertia severity, such as time of day (Dinges et al., 1985; Scheer et al., 2008), prior sleep restriction (Dinges et al., 1985; Tassi et al., 2006; McHill et al., 2017, 2019), and waking from slow wave sleep (Feltin and Broughton, 1968; Wilkinson and Stretton, 1971; Tassi et al., 1992). Research should therefore separately investigate exercise upon waking under the following conditions: (a) waking at different times of the day, including during the night when sleep inertia is typically most severe (Scheer et al., 2008); (b) waking after a period of sleep restriction; and (c) waking from different stages of sleep, particularly slow wave sleep. Knowing how these factors influence the effectiveness of exercise as a sleep inertia countermeasure could help determine the conditions in which exercise may or may not benefit on-call workers and, therefore, when to recommend exercise in the field.

#### Combining Exercise With Other Sleep Inertia Countermeasures

Future research should also investigate the effect of combining exercise with other strategies which have shown promise in countering sleep inertia. Caffeine, for example, has previously been shown to successfully reduce sleep inertia, with improvements to objective performance found at (but not before) 15 min post-waking (Hayashi et al., 2003). While this delayed benefit may not be effective in all on-call situations, combining both exercise and caffeine ingestion upon waking could potentially lead to faster and greater benefits to performance than just one strategy used in isolation. Research should therefore compare the effects of exercise alone on sleep inertia, with a separate condition in which exercise and another strategy are combined.

#### Inclusion of Subjective and Objective Measures

Subjective and objective measures of sleep inertia should be employed to compare how exercise impacts how people feel upon waking and how they perform upon waking. Inclusion of both types of measures is important since a mismatch between subjective and objective performance in response to exercise may lead to detrimental outcomes for workers in the field. For example, if workers feel more alert after exercise but do not objectively perform any better, they may think that they are performing optimally and may take more risks than if they felt less alert. Comparatively, if workers do not feel more alert after exercise but perform objectively better, then they may discount the efficacy of using exercise as a sleep inertia countermeasure. Sleep inertia is typically objectively measured using cognitive performance tasks including psychomotor vigilance tasks, reaction time tasks, digit symbol substitution tasks, addition and subtraction tasks, descending subtraction tasks, and spatial configuration tasks (Tassi and Muzet, 2000; Trotti, 2016). Some common subjective measures of sleep inertia include the Karolinska Sleepiness Scale, Stanford Sleepiness Scale, and Visual Analog Scales of alertness or sleepiness (Tassi and Muzet, 2000; Trotti, 2016).

#### Potential Effect of Exercise on Subsequent Sleep

Given that on-call personnel may return to sleep after completing a call-out and also be required to perform their jobs the next day, ensuring that their sleep is not negatively impacted by exercise when countering sleep inertia is important to protect next-day safety and performance. Exercise has been shown to have phase-shifting properties (Buxton et al., 2003; Youngstedt et al., 2019) which may have repercussions for sleep. Further, while a short bout of exercise on waking is unlikely to impact subsequent sleep later in the night, this has not been confirmed objectively to date. Future research should therefore include measures of sleep after the exercise is performed as well as a measure of circadian phase to determine whether subsequent sleep and circadian phase are impacted by the exercise.

#### Impact of Interindividual Differences on Sleep Inertia

Research into how interindividual differences impact sleep inertia is limited. Preliminary research suggests that those with later chronotypes experience sleep inertia of a longer duration than those with early chronotypes (Ritchie et al., 2017) and older adults can experience sleep inertia of a greater severity than young adults (Silva and Duffy, 2008). While no studies have directly investigated the effects of gender on sleep inertia, sex differences in chronotype (Duarte et al., 2014) and circadian factors (Santhi et al., 2016) may, in turn, affect sleep inertia. Further, it is known that the timing of the CAR peak differs between males and females, with the CAR peaking at 30 min post-waking for males, compared to 45 min post-waking for females (Stalder et al., 2016). Given that the CAR may be a driver of sleep inertia, this further demonstrates the possible influences of sex-based differences on sleep inertia. As a result, future research might consider controlling for these factors (chronotype, age, and sex) when testing the efficacy of exercise on countering sleep inertia.

#### Influence of Type of Waking

The effectiveness of exercise as a sleep inertia countermeasure may also be influenced by the type of awakening. For example, research has found that sleep inertia is more severe when waking is forced (e.g., by alarms), compared to self-awakening (Ikeda and Hayashi, 2010). In contrast, firefighters have reported they experience an “adrenaline rush” that increases alertness when waking to a confirmed call. It is possible that not only the type of awakening but also the events that follow the awakening may impact sleep inertia. Indeed, waking to an alarm under simulated on-call conditions can produce a greater CAR than being woken gently (Hall et al., 2016a). Given that on-call personnel can sometimes be required to respond to less critical events (e.g., firefighters responding to false alarms), it may be important to determine the effectiveness of exercise as a sleep inertia countermeasure in response to different types of waking (abrupt waking compared to self-awakening) and different types of events (highly critical events compared to less critical events).

### Practical Considerations for Workers

Further research is needed into the practical considerations of utilizing exercise as a sleep inertia countermeasure in the workplace. Qualitative methods, including interviews, focus groups, and surveys with on-call workers could be used to gain a better understanding of practical aspects. For example, interviews with workers could reveal the exact amount of time that workers have between receiving and responding to a call. This information could inform how long is practical for workers to exercise upon waking, and experimental investigations should be adjusted accordingly. Conducting qualitative studies in the field may also allow researchers to determine any operational or procedural barriers, such as the physical constraints of the workplace. This could inform the type or method of exercise which would suit workers. For example, if there is no room for equipment, then the effects of exercise performed without equipment on sleep inertia should be a focus of experimental investigations. To date, there have been few qualitative, field-based studies with on-call workers on the topic of sleep inertia. It is, however, vital that such research is conducted to determine how to best implement exercise, as well as other sleep inertia countermeasures, into the workplace and to facilitate translation from research to practice. Without this evidence, implementation of recommendations is likely to be impaired by environmental, occupational, and personal constraints.

## Conclusion

Strategies to effectively and efficiently reduce the impact of sleep inertia on the safety and productivity of on-call workers are needed. Currently advocated sleep inertia countermeasures are not able to dissipate sleep inertia within 15 min post-waking or have not been practical for on-call workers. A short burst of exercise upon waking is yet to be systematically investigated; however, research suggests that exercise may speed up the physiological changes that occur upon waking, which are thought to influence sleep inertia. Experimental investigations are required to isolate the effect of exercise on sleep inertia and the physiological processes which occur upon waking and to determine the exercise intensity and duration required to reduce sleep inertia. Studies are also needed to investigate the effect of exercise on sleep inertia under conditions in which it can be more severe. Finally, if exercise is found to be a successful sleep inertia countermeasure, research should be conducted to determine how to practically implement exercise in the workplace.

## Author Contributions

All authors contributed to the writing and revising of the manuscript, and read and approved the submitted version. KK wrote the first draft and revised subsequent drafts. GV, SF, JP, and BA provided critical feedback on the first draft. CH and AR contributed perspectives on their specific areas of expertise.

## Conflict of Interest

The authors declare that the research was conducted in the absence of any commercial or financial relationships that could be construed as a potential conflict of interest.
